# Paradoxical activation of transcription factor SREBP1c and *de novo* lipogenesis by hepatocyte-selective ATP-citrate lyase depletion in obese mice

**DOI:** 10.1016/j.jbc.2022.102401

**Published:** 2022-08-18

**Authors:** Batuhan Yenilmez, Mark Kelly, Guo-Fang Zhang, Nicole Wetoska, Olga R. Ilkayeva, Kyounghee Min, Leslie Rowland, Chloe DiMarzio, Wentao He, Naideline Raymond, Lawrence Lifshitz, Meixia Pan, Xianlin Han, Jun Xie, Randall H. Friedline, Jason K. Kim, Guangping Gao, Mark A. Herman, Christopher B. Newgard, Michael P. Czech

**Affiliations:** 1 Program in Molecular Medicine, University of Massachusetts Chan Medical School, Worcester, Massachusetts, USA; 2 Sarah W. Stedman Nutrition and Metabolism Center and Duke Molecular Physiology Institute, Duke University Medical Center, Durham, North Carolina, USA; 3 Department of Pharmacology and Cancer Biology, and Department of Medicine, Endocrinology and Metabolism Division, Duke University Medical Center, Durham, North Carolina, USA; 4 Department of Medicine, University of Texas Health Science Center at San Antonio, San Antonio, Texas, USA; 5 Viral Vector Core, University of Massachusetts Medical School, Worcester, Massachusetts, USA

**Keywords:** ACLY, metabolomics, liver metabolism, *de novo* lipogenesis, NAFLD, lipid metabolism, AAV, adeno-associated virus, ACC1, AcCoA carboxylase 1, AcCoA, acetyl CoA, ACLY, ATP-citrate lyase, ACSS2, AcCoA synthase short-chain family member 2, DNL, *de novo* lipogenesis, ELOVL6, elongation of long-chain fatty acid family member 6, FASN, fatty acid synthase, HFD, high-fat diet, IS, internal standard, LKO, liver KO, LKD, liver knockdown, MalCoA, malonyl CoA, MS, mass spectrometry, mTOR, mammalian target of rapamycin, NASH, nonalcoholic steatohepatitis, SCD1, stearoyl-coenzyme A desaturase 1, SREBP1c, sterol regulatory element–binding protein 1c, TBDMS, *N*-*t*-butyldimethylsilyl-*N*-methyltrifluoroacetamide, TBG, thyroxine-binding globulin, TG, triglyceride

## Abstract

Hepatic steatosis associated with high-fat diet, obesity, and type 2 diabetes is thought to be the major driver of severe liver inflammation, fibrosis, and cirrhosis. Cytosolic acetyl CoA (AcCoA), a central metabolite and substrate for *de novo* lipogenesis (DNL), is produced from citrate by ATP-citrate lyase (ACLY) and from acetate through AcCoA synthase short chain family member 2 (ACSS2). However, the relative contributions of these two enzymes to hepatic AcCoA pools and DNL rates in response to high-fat feeding are unknown. We report here that hepatocyte-selective depletion of either ACSS2 or ACLY caused similar 50% decreases in liver AcCoA levels in obese mice, showing that both pathways contribute to the generation of this DNL substrate. Unexpectedly however, the hepatocyte ACLY depletion in obese mice paradoxically increased total DNL flux measured by D_2_O incorporation into palmitate, whereas in contrast, ACSS2 depletion had no effect. The increase in liver DNL upon ACLY depletion was associated with increased expression of nuclear sterol regulatory element–binding protein 1c and of its target DNL enzymes. This upregulated DNL enzyme expression explains the increased rate of palmitate synthesis in ACLY-depleted livers. Furthermore, this increased flux through DNL may also contribute to the observed depletion of AcCoA levels because of its increased conversion to malonyl CoA and palmitate. Together, these data indicate that in fat diet–fed obese mice, hepatic DNL is not limited by its immediate substrates AcCoA or malonyl CoA but rather by activities of DNL enzymes.

One of the debilitating comorbidities of type 2 diabetes in obesity is nonalcoholic steatohepatitis (NASH), characterized by severe liver inflammation and fibrosis that can lead to cirrhosis and the need for liver transplantation (1, 2, 3, 4, 5, 6, 7, 8). NASH is driven by several cell types within the liver, including proinflammatory cells and stellate cells that secrete collagens and other extracellular matrix proteins that lead to fibrosis (3, 4, 5, 6, 7, 8, 9, 10, 11, 12). Development of hepatic steatosis often precedes NASH, associated with increased rates of fatty acid synthesis (*de novo* lipogenesis [DNL]) and lipid sequestration within lipid droplets (13, 14, 15, 16, 17). Based on the identification of polymorphisms in genes such as patatin-like phospholipase domain–containing protein 3 (PNPLA3), known to not only regulate lipid metabolism but also associated with progression of NASH, it is thought that hepatic steatosis is an important therapeutic target pathway for alleviating NASH (18, 19, 20, 21). Enzyme targets for limiting hepatic steatosis have been proposed and investigated, including diacylglycerol acyl transferase 2 (22, 23, 24, 25, 26, 27, 28, 29), which catalyzes the final step of triglyceride (TG) synthesis, and AcCoA carboxylase 1 (ACC1) (30, 31), the enzyme that converts acetyl CoA (AcCoA) to malonyl CoA (MalCoA) in the DNL pathway. Pharmacologic suppression of these enzymes has elicited decreases in hepatic steatosis as well as some indicators of NASH in humans (24, 32). Taken together, these considerations highlight the importance of gaining a more complete understanding of the dynamics of hepatic lipid synthesis in response to high-fat diet (HFD)/high sucrose diet feeding, as well as in overt obesity and type 2 diabetes.

A central metabolic intermediate in lipid metabolism is AcCoA, which in the cytosol is the substrate for both fatty acid and cholesterol synthesis, and in mitochondria serves as an intermediate of glucose and fatty acid oxidation (33). Cytosolic AcCoA has two well studied sources—citrate, which yields AcCoA and oxaloacetate when cleaved by ACLY, and acetate, which is converted to AcCoA by ACSS2. Recent studies have demonstrated that fructose supplementation *via* the drinking water induces hepatic DNL through multiple mechanisms including induction of hepatic lipogenic enzymes, and by metabolism of fructose by the microbiome to generate acetate, which then serves as the substrate for DNL *via* its conversion to AcCoA by ACSS2 (34, 35, 36). The importance of microbial acetate as a source of fructose-derived lipogenic substrate is supported by experiments showing that KO of ACLY in mice has no impact on total rates of hepatic DNL in response to fructose feeding (34). The relative contributions of ACLY and ACSS2 to AcCoA production and DNL flux have not been studied in the context of the most common experimental model of obesity, its induction by feeding with HFD. In humans, inhibition of ACLY by the small-molecule drug bempedoic acid decreases blood lipids without decreasing liver fat, but combination therapies that simultaneously target ACLY and ACSS2 have not been reported (37, 38, 39).

Based on these considerations, we have performed a study of the effects of liver-specific suppression of ACLY (ACLY liver KO [LKO]) or ACSS2 (ACSS2 liver knockdown [LKD]) expression alone, or of both genes combined (double), on hepatic DNL and levels of key metabolic intermediates including AcCoA, MalCoA, and acetate in mice fed HFD. Several surprising findings emerged: (1) while hepatocyte AcCoA levels were decreased by depletion of either ACLY or ACSS2 alone, or in combination, MalCoA levels were unchanged in response to any of these maneuvers, indicating that neither ACLY nor ACSS2 are required for maintaining levels of this immediate DNL precursor in HFD-fed obese mice; (2) there is a compensatory increase in nuclear sterol regulatory element–binding protein 1c (SREBP1c) and expression of enzymes in the DNL pathway such as fatty acid synthase (FASN), stearoyl-coenzyme A desaturase 1 (SCD1), and elongation of long-chain fatty acids family member 6 (ELOVL6) when hepatic ACLY is depleted, leading to a paradoxical increase in DNL in the absence of ACLY in HFD-fed obese mice, and (3) the upregulation of DNL enzymes occurring in response to hepatic ACLY depletion is associated with a compensatory increase in ACSS2 and a corresponding decrease in circulating acetate levels. Altogether, these studies reveal unexpected features of hepatic DNL flux in obese mice and provide a framework for understanding mechanisms that link AcCoA producing enzymes to control of more distal enzymes in the DNL pathway.

## Results

**Figure 1:**
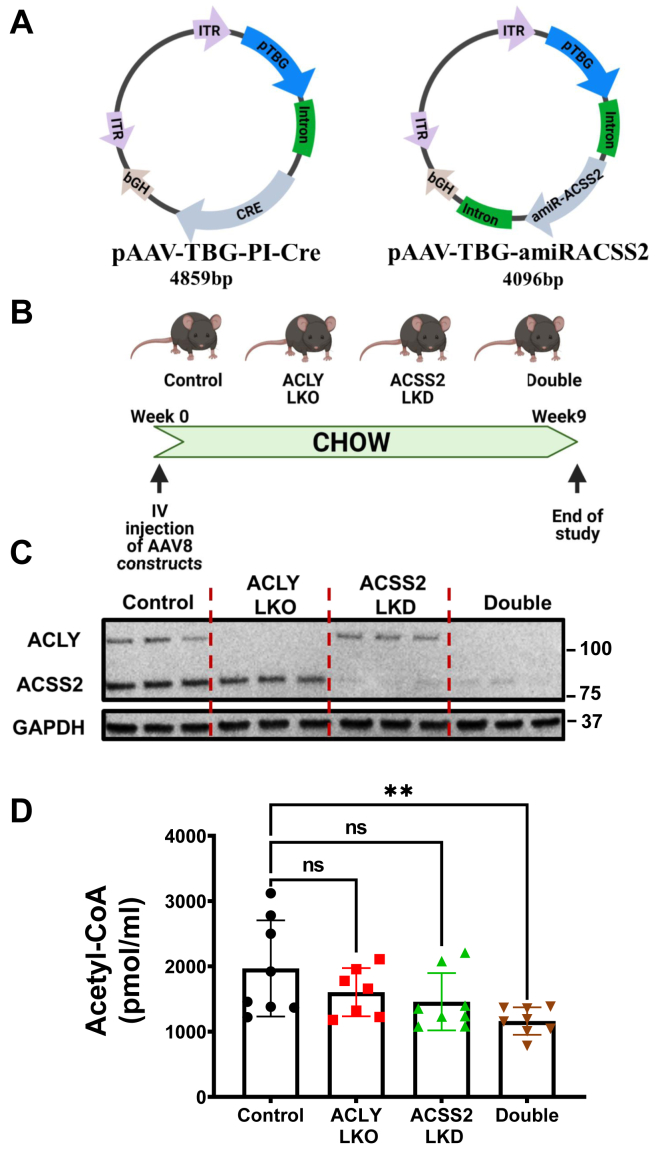
**Depletion of both ACLY and ACSS2 in hepatocytes of chow-fed mice decreases the hepatic AcCoA pool.** Eight-week-old male *Acly* fl/fl C57BL6/J mice (n = 10) fed on chow were injected IV with AAV8-TBG-PI-Cre to deplete ACLY, AAV8-TBG-amiRACSS2 to deplete ACSS2, or with both vectors to deplete both enzymes in a hepatocyte-specific manner. Nine weeks after injection, mice were sacrificed and tissues harvested. *A*, schematic rendering of viral vectors used to deplete ACLY (expressing CRE under TBG promoter) and ACSS2 (expressing artificial miRNA targeting *Acss2* mRNA under TBG promoter). *B*, study plan for investigation of hepatocyte-specific depletion of ACLY and/or ACSS2 in *Acly* fl/fl C57BL6 mice on chow diet. *C*, confirmation of lowering of ACLY and ACSS2 protein levels by immunoblotting. *D*, hepatic AcCoA levels measured by mass spectrometry (ns, not significant, ∗*p* < 0.05, ∗∗*p* < 0.005, ∗∗∗*p* < 0.0005, and ∗∗∗∗*p* < 0.00005). AcCoA, acetyl CoA; ACLY, ATP-citrate lyase; ACSS2, AcCoA synthase short-chain family member 2; IV, intravenously; TBG, thyroxine-binding globulin.

In order to determine the relative contributions of metabolic flux through ACLY *versus* ACSS2 to form AcCoA and fuel DNL in hepatocytes *in vivo*, we used mice floxed flanking exon 17 to 19 of the *acly* gene and injected with either adeno-associated virus (AAV) engineered for hepatocyte-selective Cre expression (pAAV-TBG-PI-Cre) to delete liver ACLY or AAV engineered for hepatocyte-selective expression of an artificial micro-RNA directed against ACSS2 (pAAV-TBG-amiRACSS2) to achieve liver ACSS2 depletion (Fig. 1*A*). To obtain combined depletion of ACLY and ACSS2, both AAV constructs were injected simultaneously. To establish the utility of these vectors, *acly* floxed mice fed on chow diet were injected with these AAV constructs or a control AAV and sacrificed 9 weeks later for analysis (Fig. 1*B*). Immunoblotting analysis of livers from these mice revealed the expected depletion of ACLY, ACSS2, or both proteins dependent on the vectors administered (Fig. 1*C*). In addition, immunoblots of brown, epididymal, and inguinal adipose tissue showed no decreases in ACLY or ACSS2 expression, confirming the liver specificity of the gene silencing provided by the AAV constructs (Fig. S1). No difference in body weight was noted in response to treatment with any of the AAV vectors (data not shown). Hepatic AcCoA levels did not change significantly in response to suppression of either ACLY or ACSS2 alone but were reduced ∼50% in response to combined depletion of hepatic ACLY plus ACSS2 (Fig. 1*D*). These data validate the efficacy of the AAV constructs to cause significant depletion of the targeted enzymes and demonstrate that with chow feeding, the mice are able to maintain normal hepatic AcCoA levels by alternate routes when either ACLY levels or ACSS2 levels are diminished but not when both are depleted.

**Figure 2:**
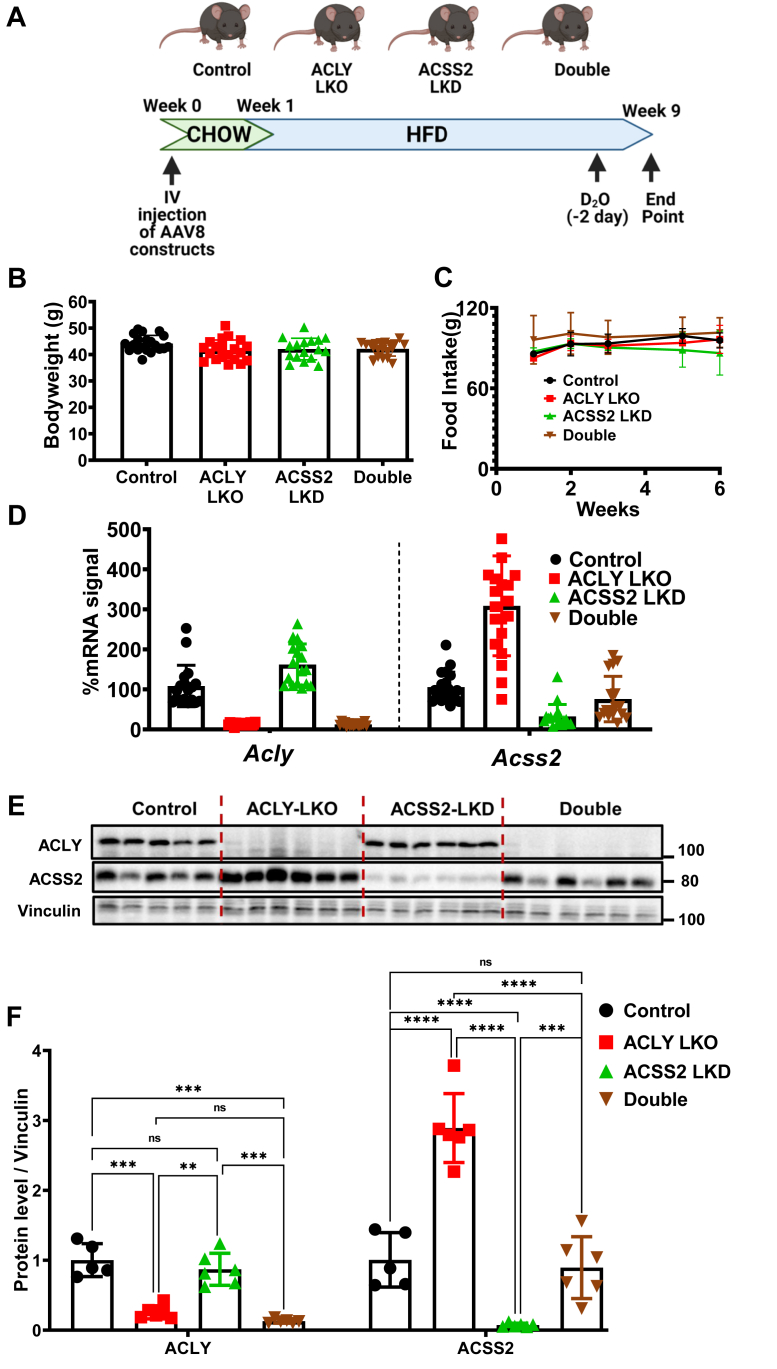
**Hepatic depletion of ACLY causes a compensatory upregulation of ACSS2 expression in mice on HFD.** Eight-week-old male *Acly* fl/fl C57BL6/J mice (n = 20) on chow were injected with viral particles *via* IV. A week after injection, mice were switched to 60% HFD. After 8 weeks of HFD feeding, mice were sacrificed and tissues harvested. Two days before the study end date, mice were injected with D_2_O (25 μl/g dose), and their drinking water was switched to 6% D_2_O to allow DNL measurements. *A*, summary of study plan. *B*, bodyweight measurements at the end of 8 weeks of HFD feeding. *C*, food intake measurements for the duration of HFD feeding. Measurements of ACLY and ACSS2 (*D*) mRNA and (*E*) protein levels in liver. *F*, quantification of ACLY and/or ACSS2 protein levels. (ns, not significant, ∗*p* < 0.05, ∗∗*p* < 0.005, ∗∗∗*p* < 0.0005, and ∗∗∗∗*p* < 0.00005). ACLY, ATP-citrate lyase; ACSS2, AcCoA synthase short-chain family member 2; DNL, *de novo* lipogenesis; HFD, high-fat diet; IV, intravenously.

Figure 2*A* depicts the experimental protocol used to address the key questions of our study concerning regulation of DNL in HFD-fed obese mice. Following injection of the AAV constructs, mice were fed chow for 1 week and then switched to a diet containing 60% fat (HFD) for eight additional weeks prior to sacrifice. No changes in body weight (Fig. 2*B*), glucose tolerance (Fig. S2), or food intake (Fig. 2*C*) were observed in mice injected with the ACLY or ACCS2 AAV vectors relative to mice injected with control AAV. Treatment of HFD-fed mice with pAAV-TBG-PI-Cre caused near complete suppression of ACLY mRNA (Fig. 2*D*) and protein (Fig. 2, *E* and *F*) levels, both when administered alone or in conjunction with the pAAV-TBG-amiRACSS2 vector that depletes ACSS2. However, in contrast to what was observed in chow-fed mice (Fig. 1), depletion of hepatocyte ACLY caused significant upregulation of ACCS2 expression. Moreover, while injection of the pAAV-TBG-amiRACSS2 vector alone caused a strong depletion of *Acss2* mRNA and protein in the obese mice, this suppression was less effective when combined with ACLY depletion, likely because of the compensatory upregulation phenomenon (Figs. 2, *D*–*F* and S3*A*). The levels of ACSS2 protein in double KO mice were similar to those in mice treated with the control AAV vector but were well below levels observed in the ACLY LKO mice.

**Figure 3:**
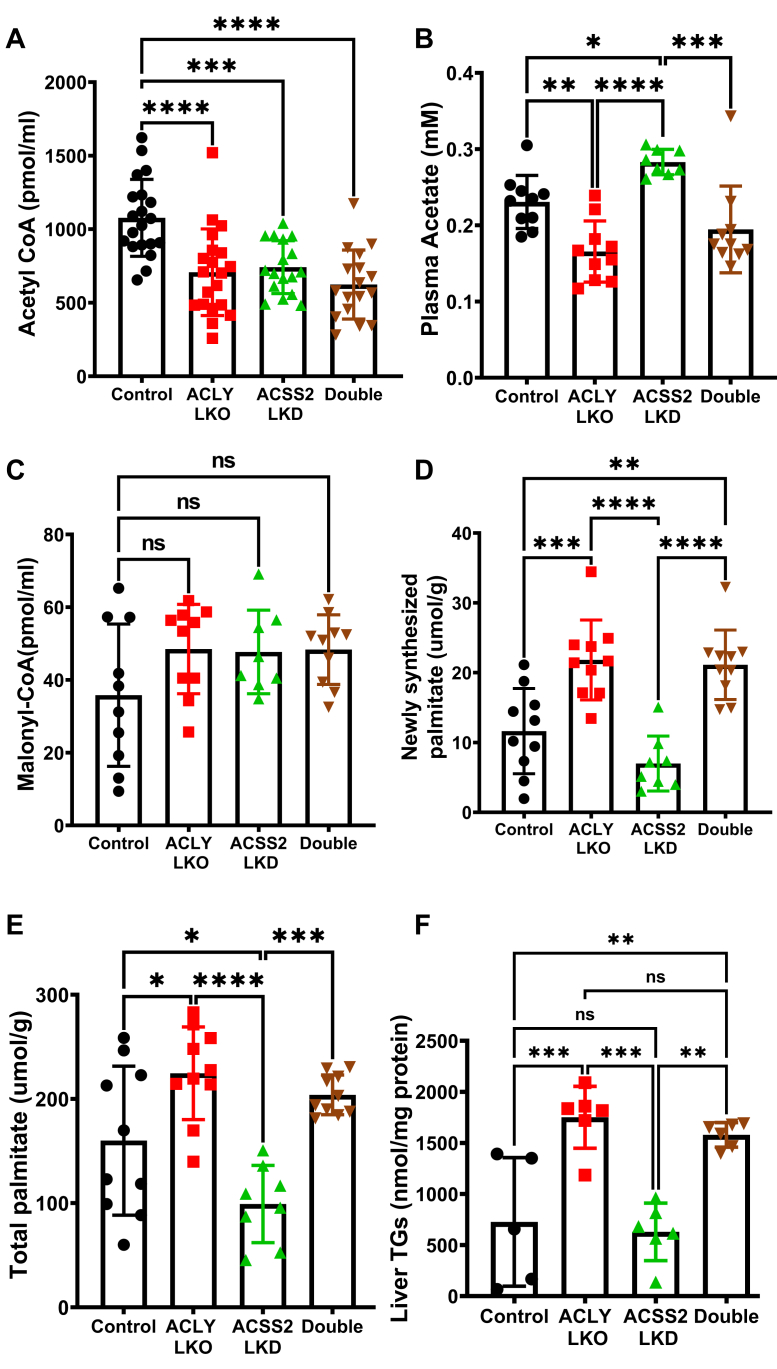
**Hepatic depletion of ACLY alone or both ACLY and ACSS2 decrease hepatic AcCoA levels but increase DNL and liver TG in mice fed HFD.** Liver homogenates were processed to measure specific metabolite changes resulting from depletion of ACLY and/or ACSS2. *A*, hepatic AcCoA levels. *B*, plasma acetate levels. *C*, hepatic MalCoA levels measured by LC–MS/MS. *D*, hepatic newly synthesized palmitate levels (measured by deuterium incorporation from D_2_O into liver palmitate). *E*, hepatic total palmitate levels. *F*, liver TG levels per milligram of protein. (ns, not significant, ∗*p* < 0.05, ∗∗*p* < 0.005, ∗∗∗*p* < 0.0005, and ∗∗∗∗*p* < 0.00005). AcCoA, acetyl CoA; ACLY, ATP-citrate lyase; ACSS2, AcCoA synthase short-chain family member 2; DNL, *de novo* lipogenesis; HFD, high-fat diet; MalCoA, malonyl CoA; TG, triglyceride.

Next, metabolite levels and metabolic flux through DNL were assessed in two separate cohorts of HFD-fed obese mice, and the results of the two studies were combined to provide the data in Figure 3. Under HFD conditions, KD of either ACLY or ACSS2 in hepatocytes led to a significant decrease in AcCoA levels, with a trend for further decline in mice with combined KD of both enzymes (Fig. 3*A*). Notably, the compensatory increase in ACSS2 expression in the ACLY LKO condition did not restore AcCoA levels in the absence of ACLY. That ACSS2 is active under these conditions is verified by analysis of plasma acetate (Fig. 3*B*), which was inversely proportional to ACSS2 expression. Notably, plasma acetate levels were also decreased in response to combined suppression of ACLY and ACSS2, suggesting that even though ACSS2 protein was not elevated in this condition compared with mice treated with the control AAV vector, flux through this enzyme and its consumption of acetate may have increased. Remarkably, the fall in AcCoA levels elicited by KD of ACLY, ACSS2, or both enzymes was not accompanied by a decrease in MalCoA levels (Fig. 3*C*). Also unanticipated, newly synthesized palmitate and total palmitate levels were increased in response to ACLY LKO, either alone or when combined with ACSS2 suppression, whereas ACSS2 LKD caused a modest decrease in these outcomes, despite the decrease in AcCoA levels in response to all these maneuvers (Fig. 3, *B D*, and *E*).

Total liver TG levels also followed the newly synthesized palmitate and total palmitate trend. Hepatic TG levels were significantly increased by ACLY depletion or double depletion compared with the ACSS2 LKD and control group (Fig. 3*F*). None of these changes in hepatic lipid biosynthesis were reflected in changes in plasma lipids (Fig. S4). These data indicate that AcCoA levels do not determine MalCoA levels or rates of DNL under HFD-fed conditions in mice. Instead, the metabolite that most strongly correlated, in an inverse fashion, with DNL was plasma acetate, which was reduced in response to ACLY depletion (alone or in concert with ACSS2 KD) and slightly increased in response to ACSS2 LKD alone.

**Figure 4:**
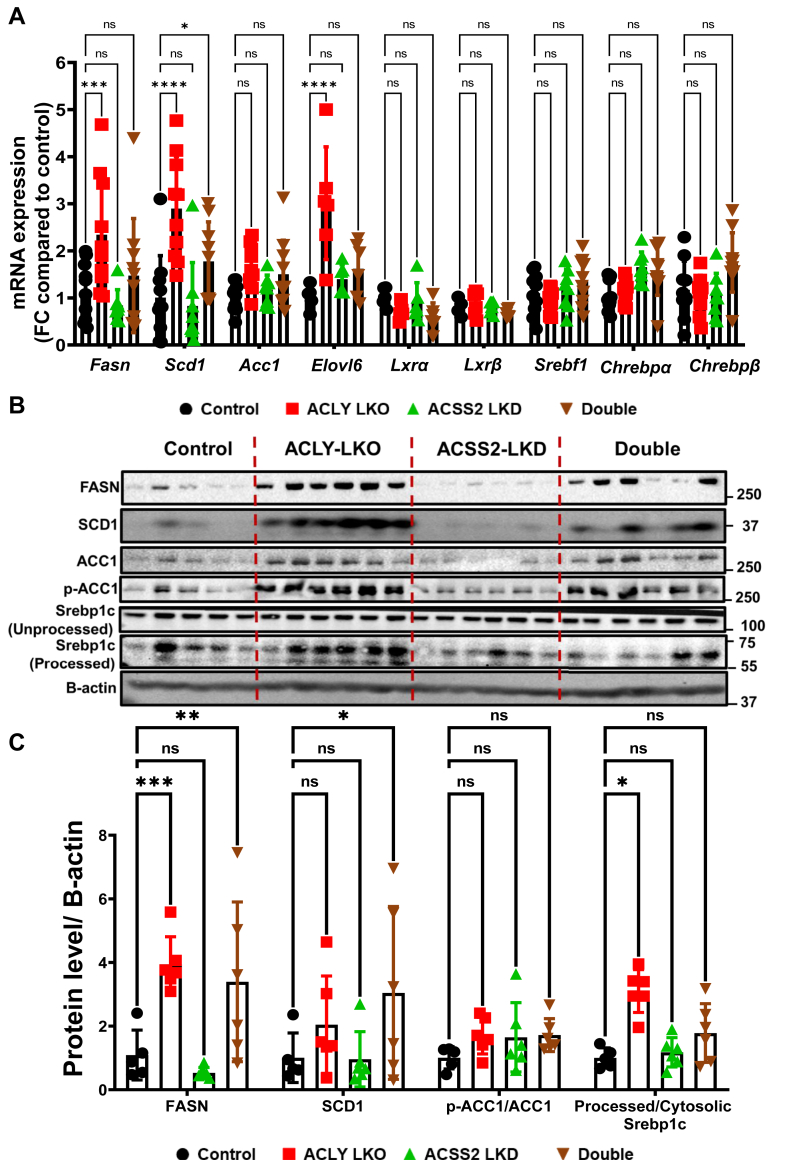
**Hepatic depletion of ACLY but not of ACSS2 results in upregulation of enzymes in the DNL pathway processed (active) SREBP1c protein levels.** Livers from the HFD-fed mouse groups were processed for investigation of (*A*) mRNA and (*B*) protein expression changes between the groups. *C*, quantification of protein levels between groups. (ns, not significant, ∗*p* < 0.05, ∗∗*p* < 0.005, ∗∗∗*p* < 0.0005, and ∗∗∗∗*p* < 0.00005). ACLY, ATP-citrate lyase; ACSS2, AcCoA synthase short-chain family member 2; DNL, *de novo* lipogenesis; HFD, high-fat diet; SREBP1c, sterol regulatory element–binding protein 1c.

We next explored the mechanisms underlying the paradoxical increase in DNL engendered by ACLY LKO, occurring in the face of lowered AcCoA levels and unchanged MalCoA levels. To this end, we measured expression of the genes encoding key enzymes in the DNL pathway as well as transcription factors known to control their expression (Fig. 4). At the mRNA level, three enzymes in the DNL pathway were found to be significantly elevated in ACLY-depleted livers—*Fasn*, *Scd1*, and *Elovl6* (Fig. 4*A*). Interestingly, this upregulation has been detected in only HFD-fed obese mice but not in lean chow-fed mice (Fig. S5). In contrast, ACSS2 depletion alone, which had no significant effect on newly synthesized palmitate (Fig. 3*D*), also had no effect on *Fasn*, *Scd1*, or *Elovl6* mRNA levels (Fig. 4*A*). Immunoblot analyses confirmed the increase in FASN and SCD1 at the protein level and also revealed an increase in ACC1, both total amount and its phosphorylated inactivated form in the ACLY LKO condition, resulting in no net change in the inactive to active ACC1 ratio (Fig. 4, *B* and *C*). Interestingly, depletion of ACSS2 in addition to ACLY LKO abrogated the increase in DNL enzyme mRNA levels but not protein levels. These data suggest that hepatic DNL in HFD-fed obese mice, but not in chow-fed (Fig. S5) mice, is not limited by or dependent on AcCoA and MalCoA levels but rather is regulated by altered expression and activities of DNL enzymes such as FASN and SCD1.

Since it is known that the transcription factors ChREBP and SREBP1c regulate the expression of DNL enzymes in liver, we analyzed expression of these transcription factors in our genetically engineered HFD-fed mice. While no difference in expression levels of these transcription factors at the mRNA level was observed, we found a clear effect on levels of the nuclear-localized form of SREBP1c. Figures 4, *B* and *C* and S3*B* show that hepatic ACLY LKO but not ACSS2 LKD causes increased processing of SREBP1c to the nuclear form, an effect not observed when ACLY LKO was combined with ACSS2 LKD. These data suggest that ACLY depletion causes upregulation of SREBP1c processing to its activated form, associated with upregulation of ACSS2 as well as SCD1 and FASN. Although this effect is not significant when ACLY is depleted in combination with ACSS2 depletion, the trend is still evident and the DNL enzymes are upregulated in this condition (Fig. 4*C*).

**Figure 5:**
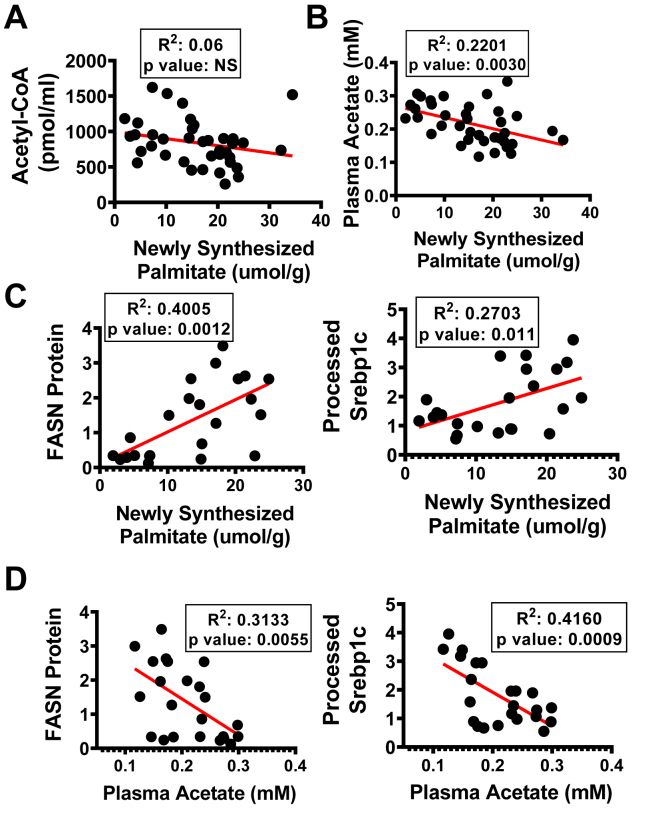
**Newly synthesized palmitate levels (DNL) and plasma acetate correlate with FASN expression levels but not AcCoA.** Correlation graphs of (*A*) AcCoA *versus* newly synthesized palmitate, (*B*) plasma acetate *versus* newly synthesized palmitate, (*C*) FASN and processed SREBP1c protein levels *versus* newly synthesized palmitate, (*D*) FASN and processed SREBP1c protein levels *versus* plasma acetate. (ns, not significant, ∗*p* < 0.05, ∗∗*p* < 0.005, ∗∗∗*p* < 0.0005, and ∗∗∗∗*p* < 0.00005). AcCoA, acetyl CoA; DNL, *de novo* lipogenesis; FASN, fatty acid synthase; SREBP1c, sterol regulatory element–binding protein 1c.

We applied correlation analyses of data from individual mice to investigate how Srebp1 processing and DNL enzyme expression may be related to the ACLY and ACSS2 perturbations (Fig. 5). This analysis confirmed the lack of correlation between AcCoA levels and newly synthesized palmitate but showed a significant correlation between plasma acetate levels and DNL (Fig. 5, *A* and *B*). Moreover, there were strong correlations between FASN protein expression (Fig. 5*C*) and SREBP1c processing (Fig. 5*C*) with DNL flux. Importantly, plasma acetate concentrations correlated inversely with FASN expression (Fig. 5*D*) and with the nuclear form of SREBP1c (Fig. 5*D*), consistent with the concept that acetate levels are an indicator of SREBP1c activity, hepatic DNL enzyme expression, and hepatic DNL flux.

**Figure 6:**
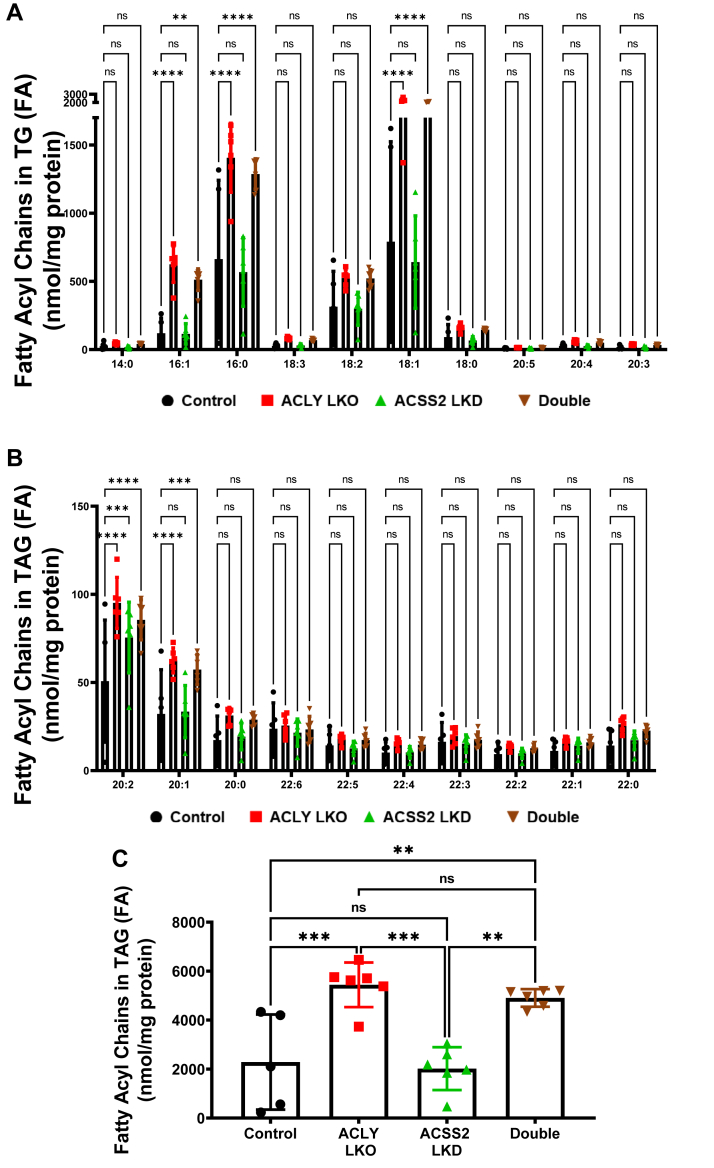
**Lipidomics analysis of individual fatty acid species in TGs.***A* and *B*, measurements of individual fatty acid species in TGs. *C*, measurements of total liver fatty acyl chains in TGs in the livers of HFD-fed groups. (ns, not significant, ∗*p* < 0.05, ∗∗*p* < 0.005, ∗∗∗*p* < 0.0005, and ∗∗∗∗*p* < 0.00005). HFD, high-fat diet; TG, triglyceride.

In addition, lipidomics analysis showed significantly increased diglyceride accumulation (Fig. S6*A*) as well as fatty acyl chains in TGs in ACLY LKO and double depletion groups compared with ACSS2 LKD and control groups (Fig. 6, *A–C*). In line with the DNL increase in ACLY-depleted groups (Fig. 3*D*), the major individual fatty acid species in TGs were also increased in ACLY-depleted groups (Fig. 6, *A–C*). Interestingly, the increase in newly synthesized palmitate, liver TG, and diglyceride accumulation was inversely correlated with phospholipid biosynthesis, suggesting that DNL is shunted toward TG biosynthesis compared with phospholipid biosynthesis under conditions where ACLY is absent (Fig. S6, *B*–*D*).

## Discussion

A primary question addressed in this study is the degree to which the two AcCoA synthesis pathways catalyzed by ACLY *versus* ACSS2 contribute to steady-state levels of AcCoA in livers of HFD-fed obese mice. The importance of this question is reinforced by several perspectives: (1) AcCoA is a major substrate in the pathways of DNL and cholesterol synthesis, both of which are upregulated in obesity to contribute to metabolic disease, (2) AcCoA is a substrate for histone and other protein acetylation reactions that control gene expression and enzyme function, and (3) effects of single and double depletions of hepatocyte ACLY and ACSS2 on levels of key DNL intermediates such as AcCoA, MalCoA, and acetate, and flux through the DNL pathway, have not been investigated in the most commonly used animal model of obesity, the HFD-fed mouse. To perform these experiments in the most rigorous manner, AAV vectors were employed expressing thyroxine-binding globulin (TBG) promoter–based constructs to assure hepatocyte-selective depletion of ACLY and ACSS2 (Fig. 1). The results indicate that ACLY and ACSS2 both contribute significantly to AcCoA levels in hepatocytes of HFD-fed obese mice, as evidenced by significant decreases in content following depletion of each enzyme alone or both in combination (Figs. 2, 3 and S1). In contrast, in mice on chow diet, hepatic AcCoA levels were unchanged in response to suppression of either ACLY or ACSS2 alone and declined only when both ACLY and ACSS2 were suppressed (Fig. 1*D*). These data show that HFD-fed obese mice, unlike lean mice, require both ACLY and ACSS2 to maintain their normal hepatic AcCoA levels.

It is important to note that there are some limitations in the interpretations that we can make in these studies. Even with the double depletion of ACLY and ACSS2 in hepatocytes, no more than a 50% decrement in hepatic AcCoA was observed in either the chow-fed mice or HFD-fed mice (Figs. 1 and 3). In obese mice, the partial decrease in AcCoA might be explained by the lack of complete elimination of ACSS2 in the double depletion condition (Fig. 2*E*), although this does not explain the modest decrease in AcCoA levels in chow-fed mice with complete suppression of both hepatic enzymes (Fig. 1*C*). There are several possible reasons for this modest decrease in the measured AcCoA levels when both enzymes are missing. First, other pathways of cytosolic AcCoA generation may be at play. For example, it has been proposed that cytosolic AcCoA can be regenerated from acetylcarnitine by a putative cytosolic carnitine acetyltransferase, which has been reported to be present in mammalian tissue, albeit not yet in liver (40, 41). Second, the extracts assayed here are from whole liver and therefore include other cell types that also contain AcCoA such as endothelial cells and macrophages that are not depleted of ACLY or ACSS2, given that we specifically targeted hepatocytes through TBG promoter–driven AAV constructs. Third, cytosolic AcCoA derived from ACLY and ACSS2 represents only one compartment of AcCoA in the hepatocyte, whereas significant AcCoA pools in other cellular compartments such as mitochondria and peroxisomes are well established (41, 42, 43, 44, 45, 46, 47). In mitochondria, AcCoA is produced by glucose, fatty acid, and amino acid oxidation (42, 43, 46, 47, 48, 49, 50, 51, 52). In peroxisomes, AcCoA is also generated as a result of active fatty acid oxidation by a pathway defined by the rate-limiting enzyme ACOX1 (53). Finally, cytosolic AcCoA may be derived *via* export from the nucleus (46, 54, 55), although this pool is expected to also require ACLY and ACSS2 as does the cytosolic pool. Taken together, these considerations indicate that the roughly 50% decrease in AcCoA observed in the whole livers of HFD-fed mice depleted of ACLY or ACSS2 represent minimal estimates of the reduction in the cytosolic AcCoA pool in hepatocytes.

In the case of single ACLY LKO, a decrease of about 40% in AcCoA occurs despite a strong compensatory upregulation of ACSS2 protein to approximately three times its normal level (Fig. 2, *D* and *E*). Similar compensatory upregulation was also reported in adipocyte-specific ACLY depletion previously (56). Yet, when this ACSS2 compensation is blocked in the double KD, that is, when ACLY is depleted and ACSS2 remains at basal levels (Fig. 2, *D* and *E*), AcCoA levels are lowered to a similar extent as in the ACLY LKO alone. While this result seems to suggest that ACSS2 may not contribute much to the AcCoA levels, ACSS2 LKD alone causes a 40% decrease in AcCoA, affirming its significant contribution to the pool (Fig. 3*A*). Reconciliation of these findings may be reached by our observation that in HFD-fed obese mice, ACLY LKO causes a large increase in expression of DNL enzymes (Fig. 4) and in DNL flux to increase palmitate synthesis (Fig. 3, *D* and *E*). This increased DNL flux secondary to the upregulation of downstream DNL enzymes likely exerts an increased forward flux from the AcCoA pool (Fig. 3*A*). This occurs in addition to the presumed decreased generation of AcCoA from ACLY depletion resulting in lower total AcCoA levels in ACLY LKO mice despite the significant ACSS2 upregulation. In addition, significant increase in newly synthesized palmitate also translates to the individual fatty acid species in TG (Fig. 6, *A–C*), strengthening the idea of the increased forward-flux phenomenon throughout the DNL pathway.

Interestingly, MalCoA levels remain unchanged in the face of ACLY or ACSS2 depletion, even though utilization of this intermediate for DNL was clearly increased in ACLY LKO mice. This may be due to the dynamic nature of MalCoA in the DNL pathway that leads to conversion of MalCoA to palmitate fast enough that cells do not have prolonged high concentration of MalCoA. In this regard, the pACC1:total ACC1 ratio is unchanged, as both species are elevated, suggesting that overall ACC1 activity is not much altered in the ACLYKO condition. These data also suggest that citrate may not be playing an active role in activating ACC1 under the conditions of our experiments. Taken together, our results suggest that ACSS2 is the predominant contributor to AcCoA generation in HFD-fed obese mice since ACSS2 depletion reduces AcCoA levels to an extent equal to the ACLY LKO condition but with no change in DNL flux (Figs. 3 and 4). This concept is consistent with work indicating a major role for acetate in liver lipogenesis as recently published by others using different mouse models (34, 57, 58). Furthermore, it is consistent with the marked decrease in plasma acetate that occurs in conjunction with the upregulation of ACSS2 in the ACLY LKO condition, indicating significant flux of acetate into DNL (Fig. 3*B*). A strong correlation between the decrease in plasma acetate and increase in DNL activity (Fig. 5*B*) is also noted, further supporting the role of acetate as a major substrate for DNL in our HFD-fed mouse model of obesity.

The results described previously raise an important mechanistic question: How does hepatocyte ACLY depletion in HFD-fed obese mice result in increased expression of DNL enzymes? We initially found no changes in mRNA expression levels of two of the major transcription factors that regulate enzymes of lipid biosynthesis, SREBP1c (59, 60, 61) and ChREBP (62, 63, 64, 65). However, further investigation revealed a clear increase in levels of the processed, nuclear-localized, and active form of SREBP1c protein in livers of obese ACLY LKO mice (Figs. 4*B* and S3*B*). Interestingly, although it is known that nuclear SREBP1c protein can induce its own gene expression by binding the sterol response element region in its own promoter (66), we did not detect an increase in *Srebf1* gene expression (Fig. 4*A*). This might be due to possible lesser affinity to the *Srebf1* promoter under insulin-insensitive HFD conditions in liver. The increase in mature SREBP1c levels strongly correlated with DNL rate (Fig. 5*C*). Thus, our results suggest that ACLY LKO in hepatocytes of obese mice causes proteolytic processing of SREBP1c to its transcriptionally active form through an unknown mechanism. Two pathways known to modulate SREBP1c are mammalian target of rapamycin (mTOR) complex 1 (67, 68, 69) and AMP-activated protein kinase (57, 70). However, we did not observe a change in mTOR complex 1 activity in response to ACLY LKO, as measured by levels of p70S6K or p4-EBP (Fig. S7*A*). We also were unable to detect significant changes in levels of phosphorylated AMP-activated protein kinase levels in the ACLY LKO condition (Fig. S7*B*). And further, we have investigated the expression of Insig1 and Insig2, well-known inhibitors of SREBP1c processing (71, 72). We have not observed any reduction of Insig2a, Insig2b, or Insig1 gene expression in the ACLY-depleted groups on HFD (Fig. S7*C*). Another possible explanation to increased SREBP1c processing is through SCD1–oleate axis as reported previously (73, 74). Although we did not detect significant changes in activation of canonical mTOR substrates such as 4EBP and p70S6 kinase (74), this pathway still could be an explanation because of our HFD (Research Diets; catalog no.: D12492) being rich in palmitate (C16:0), stearate (C18:0), and most importantly, oleate (C18:1). ACLY depletion might be affecting the utilization of dietary oleate in an unknown way to induce the activation of SREBP1c processing. Thus, the mechanism connecting ACLY expression and regulation of SREBP1c processing will require further investigation.

It is noteworthy that studies in other mouse model systems have not revealed a connection between ACLY LKO and increased DNL activity that we report here in the HFD-obese mouse model. Wang *et al.* (75) used adenovirus-shRNA to deplete ACLY in db/db mice (a genetic model of obesity) but fed the mice chow rather than HFD. In that setting, depletion of ACLY caused a reduction in total liver AcCoA, similar to our findings in HFD-obese mice (Fig. 3), but contrary to our findings, they also reported reduced DNL as measured by D_2_O incorporation into newly synthesized palmitate, reduced liver TGs, and reduced expression of DNL enzymes in the ACLY KD condition (75). On the other hand, Zhao *et al.* (34) used mice on a high fructose diet to study ACLY and ACSS2 depletion in liver and reported that fructose-mediated lipogenesis is driven by acetate produced in the gut (34). When labeled glucose was used to measure DNL rates in that study, depletion of ACLY abolished DNL, as expected since glucose carbons must pass through ACLY to promote DNL. However, when D_2_O was used for measurements of DNL, no effect of ACLY depletion was observed, in contrast with the marked increase we reported here (34). Finally, while our article was being revised, an article published by Marrow *et al.* (76) showed increased DNL gene expression, similar to our findings but surprisingly decreased glucose-driven DNL and contrary to our finding decreased liver TG content in ACLY LKO mice on Western diet supplemented by fructose in drinking water. They also reported increased fatty acid oxidation, which might be the reason for the lower liver TG rather than the glucose-driven DNL contribution. It is possible that in addition to the differences in dietary model (high fructose *versus* high fat), differences in the time course of the experiment and other study details such as their use of albumin promoter-driven Cre recombinase to elicit ACLY depletion in the study by Zhao *et al*., and the extent of compensatory ACSS2 induction in both models, may help to explain the contrasting results.

Although data in humans at the level of detail described in our studies are not available, the ACLY inhibitor bempedoic acid has been used clinically in human subjects (38, 77, 78, 79, 80, 81). In phase 3 clinical trials, bempedoic acid reduced the mean low-density lipoprotein cholesterol level by up to 30% (81) in patients with hypercholesterolemia and by around 18% in patients on statin medications (80). Moreover, the safety and efficacy of bempedoic acid were consistently favorable following 1 year of administration (80), and Food and Drug Administration approval was obtained in February 2020. In our plasma analyses of total cholesterol (Fig. S2*A*), no differences were observed under conditions of single or double depletions of ACLY and ACSS2 compared with control groups (Fig. S2). One possible reason for difference is that bempedoic acid treatment has been shown to induce upregulation of low-density lipoprotein receptor (79), which in turn would increase the uptake of lipoproteins from the circulation. Other possible explanations for the divergent results include the species difference (mouse *versus* human) and our hepatocyte-specific deletion of ACLY in mice *versus* orally delivered bempedoic acid in human subjects that may be more widely distributed to other tissues. Bempedoic acid is converted to its bioactive form by very long-chain acyl-CoA synthase (ACSVL1), which is purported to be liver specific (38, 77, 78, 79, 80, 81). However, single-cell RNA sequencing and the human protein atlas database reveal considerable ACSVL1 mRNA and protein presence in other metabolically active tissues such as kidney and gastrointestinal tissues (82, 83, 84). Potential systemic effects as a consequence of ACLY inhibition in kidney and the gastrointestinal track remain to be investigated. Moreover, there are no data showing reduction of liver DNL in humans in response to bempedoic acid administration, and therefore, it is not yet possible to compare our results in mice to human subjects.

In summary, we demonstrate that in HFD-fed obese mice, both ACLY and ACSS2 contribute to the AcCoA pool in hepatocytes, although the ACSS2 pathway may predominate. This latter point is supported by the increase in blood acetate when ACSS2 is depleted, indicating significant backup of acetate when the ACSS2 pathway is blocked. Importantly, depletion of hepatic ACLY in these mice triggers a mechanism whereby SREBP1c is activated, and DNL-related enzymes are upregulated to increase the DNL rate. Thus, distal DNL enzymes rather than AcCoA-generating enzymes are the primary regulators of hepatic palmitate synthesis by regulating the metabolite utilization *via* the increased forward flux from the AcCoA pool to DNL in obese mice during HFD feeding.

## Experimental procedures

### Animal studies

Animal experiments were performed in accordance with animal care ethics approval and guidelines of University of Massachusetts Medical School Institutional Animal Care and Use Committee (protocol number: A-1600-19). For *in vivo* studies, *acly* floxed mice were purchased from Taconic Biosciences. Eight-week-old, male, *Acly* floxed mice were injected with corresponding AAV8 constructs *via* intravenous injection to achieve hepatocyte-specific depletion of ACLY and/or ACSS2. One week after the administration of the AAV8 constructs, these mice either stayed on standard chow or were switched to HFD for 8 weeks. Two days prior to the end point of the study, mice were prebled and then injected with 25 μl/g of bodyweight D_2_O along with supplementation of 6% D_2_O into the drinking water. Blood samples were taken at 1 day prior to the end point as well as terminally for measurement of D_2_O enrichment in the circulation.

### Generation of hepatocyte-specific AAV8 construct

The artificial miRNA against mouse Acss-2 was designed as previously described (85), and the synthesized gBlock was incorporated after the liver-specific TBG promoter in pAAV-TBG-PI vector plasmid (86) by Gibson assembly. rAAV8 was produced by transient humane embryonic kidney 293 cell transfection and CsCl sedimentation by the University of Massachusetts Medical School Viral Vector Core, as previously described (87). Vector preparations were determined by droplet digital PCR, and purity was assessed by 4 to 12% SDS-acrylamide gel electrophoresis and silver staining (Invitrogen).

### RNA isolation and RT–quantitative PCR

Frozen liver tissue punches (25–50 mg) were homogenized in TRIzol using the Qiagen TissueLyser II. Chloroform was added to the homogenate and centrifuged for 15 min at maximum speed. The clear upper layer was added to an equal volume of 100% isopropanol and incubated for 1 h at 4 °C. After 10 min of centrifugation at maximum speed, the supernatant was discarded and 70 to 75% ethanol was added to wash the pellet. After 15 min of centrifugation at maximum speed, the supernatant was discarded, and the pellet was briefly dried in the hood before being resuspended in double-distilled water. RNA concentration was then measured by the Thermo Scientific NanoDrop2000 spectrophotometer. Complementary DNA was synthesized from 1 μg of total RNA using iScript cDNA Synthesis Kit (Bio-Rad) and Bio-Rad T100 thermocycler. Quantitative RT–PCR was performed using iQ SybrGreen Supermix on the Bio-Rad CFX96 C1000 Touch Thermal Cycler and analyzed as previously described (88).

### Immunoblotting

For protein expression analyses, frozen liver tissue (∼25 mg) was homogenized by the Qiagen TissueLyser II in a sucrose buffer (250 mM sucrose, 50 mM Tris–Cl, pH 7.4) with 1:100 protease inhibitor (Sigma–Aldrich). The tissue lysates were denatured by boiling, separated on a 4 to 15% SDS/polyacrylamide gel electrophoresis gel (Bio-Rad), and transferred to a nitrocellulose membrane (Bio-Rad). The membrane was blocked with TBS-T containing 5% milk for 1 h at room temperature and incubated with primary antibodies; ACLY, ACSS2, FASN, SCD1, ACC1, p-ACC1, GAPDH, β-actin purchased from Cell Signaling, or SREBP1c antibody purchased from Millipore. The blot was washed in TBS-T for an hour, incubated at room temperature with corresponding second antibody at room temperature for 30 min, washed again, and incubated with ECL (PerkinElmer) and visualized with the ChemiDox XRS+ image-forming system.

### Lipidomics analysis

Lipid species in liver samples were analyzed using multidimensional mass spectrometry (MS)–based shotgun lipidomic analysis (89). In brief, each liver tissue sample homogenate containing 0.5 mg of protein (determined with a Pierce bicinchoninic acid assay) was accurately transferred to a disposable glass culture test tube. A premixture of lipid internal standard (IS) was added prior to conducting lipid extraction for quantification of the targeted lipid species. Lipid extraction was performed using a modified Bligh and Dyer procedure, and each lipid extract was reconstituted in chloroform:methanol (1:1, v:v) at a volume of 400 μl/mg protein (90).

For shotgun lipidomics, the lipid extract was further diluted to a final concentration of ∼500 fmol total lipids per microliter. Mass spectrometric analysis was performed on a triple quadrupole mass spectrometer (TSQ Altis; Thermo Fisher Scientific) and a Q Exactive mass spectrometer (Thermo Fisher Scientific), both of which were equipped with an automated nanospray device (TriVersa NanoMate; Advion Bioscience Ltd) as described (91). Identification and quantification of lipid species were performed using an automated software program (92). Data processing (*e.g.*, ion peak selection, baseline correction, data transfer, peak intensity comparison, and quantitation) was performed as described (92). The results were normalized to the protein content (nanomole lipid/milligram protein).

### Metabolomics

Liver acyl CoA esters were analyzed as previously described (93, 94) by flow injection analysis using positive electrospray ionization on Xevo TQ-S, triple quadrupole mass spectrometer (Waters) employing methanol/water (80/20, v/v) containing 30 mM ammonium hydroxide as the mobile phase. Spectra were acquired in the multichannel acquisition mode monitoring the neutral loss of 507 amu. Heptadecanoyl CoA was employed as an IS. The endogenous CoAs were quantified using calibrators prepared by spiking liver homogenates with authentic CoAs (Sigma) having saturated acyl chain lengths C2–C18. Corrections for the heavy isotope effects, mainly ^13^C, to the adjacent m + 2 spectral peaks in a particular chain length cluster were made empirically by referring to the observed spectra for the analytical standards.

MalCoA was extracted with 0.3 M perchloric acid and analyzed by LC–MS/MS using a previously published method (95). The extracts were spiked with ^13^C_2_-AcCoA (Sigma), centrifuged, and filtered through Millipore Ultrafree-MC 0.1 μm centrifugal filters before being injected onto a Chromolith FastGradient RP-18e HPLC column, 50 × 2 mm (EMD Millipore) and analyzed on a Waters Xevo TQ-S triple quadrupole mass spectrometer coupled to a Waters Acquity UPLC system (Waters).

### DNL measurements

DNL was measured as previously described (49). Total palmitic acid labeling assay in the liver was assayed by GC–MS. Briefly, 20 mg liver tissue was homogenized in 1 ml KOH/EtOH (EtOH 75%) and incubated at 85 °C for 3 h, and 200 μl of 1 mM [^13^C_16_]palmitate was added to samples as IS after cool down. Extracted palmitate acid was mixed with 50 μl *N*-*t*-butyldimethylsilyl-*N*-methyltrifluoroacetamide (TBDMS) at 70 °C for 30 min, and the TBDMS-derivatized samples were analyzed with an Agilent 5973N-MSD equipped with an Agilent 6890 GC system, and a DB-17MS capillary column (30 m × 0.25 mm × 0.25 μm). The mass spectrometer was operated in the electron impact mode (70 eV). The temperature program was as follows: 100 °C initial, increase by 15 °C/min to 295 °C, and hold for 8 min. The sample was injected at a split ratio of 10:1 with a helium flow of 1 ml/min. Palmitate–TBDMS derivative eluted at 9.7 min, and the *m/z* at 313, 314, and 319 were extracted for M0, M1, and M16 palmitate quantification.

Stable isotope labeling was corrected for natural isotope abundance (96). Newly synthesized palmitic acid was calculated as: percent of newly synthesized palmitic acid labeling = total palmitic acid labeling/(plasma ^2^H_2_O labeling × 22) × 100.

### Plasma acetate measurements

Acetate in plasma was quantified by LC–MS/MS method as described (97, 98), with modifications. About 30 μl plasma was mixed with 30 μl of 200 μM [^2^H_5_]acetate as IS. Acetonitrile (1 ml) was added, and the sample was centrifuged for 20 min at 8000*g* to remove protein. The supernatant was transferred to a new Eppendorf vial and was dried completely under nitrogen gas. The dried residue was derivatized with 20 μl 120 mM 1-ethyl-3-(3-dimethylaminopropyl)carbodiimide hydrochloride, 20 μl 200 mM 3-nitrophenylhydrazine hydrochloride, and 50 μl LC–MS grade water at 40 °C for 30 min. The sample was subjected to LC–MS/MS analysis, with ionization and fragmentation of acetate/[^2^H_5_]acetate optimized in negative electrospray ionization by QTRAP 6500^+^-MS (Sciex). A gradient LC method was developed with an Agilent Pursuit XRs 5 C18 column (150 × 2.0 mm, 5 μm). Mobile phase A was 98% water (LC–MS grade) and 2% acetonitrile (LC–MS grade) containing 0.1% formic acid. Mobile phase B was 98% acetonitrile and 2% water containing 0.1% formic acid. MS/MS ion transitions for acetate and [^2^H_5_]acetate were *m/z* 194/151 and 199/155, respectively. Data were analyzed by SCIEX Analyst 1.6 software.

### Software and statistics

All statistical analyses were performed using the GraphPad Prism 8 (GraphPad Software, Inc). The data are presented as mean ± SEM. For analysis of the statistical significance between four or more groups, two-way ANOVA and multiple comparison *t* tests were used. NS is nonsignificant (*p* > 0.05), ∗*p* < 0.05, ∗∗*p* < 0.005, and ∗∗∗*p* < 0.0005.

## Data availability

The data that support the findings of this study are openly available upon request.

## Supporting information

This article contains supporting information.

## Conflict of interest

The authors declare that they have no conflicts of interest with the contents of this article.
