# Supplementary material for: Paradoxical activation of transcription factor SREBP1c and de novo lipogenesis by hepatocyte-selective ATP-citrate lyase depletion in obese mice
Source: J Biol Chem. 2022 Aug 18;298(10):102401. doi: 10.1016/j.jbc.2022.102401 (PMC9490592; doi:10.1016/j.jbc.2022.102401)
Supplement: Supporting Information [file mmc1.pdf]

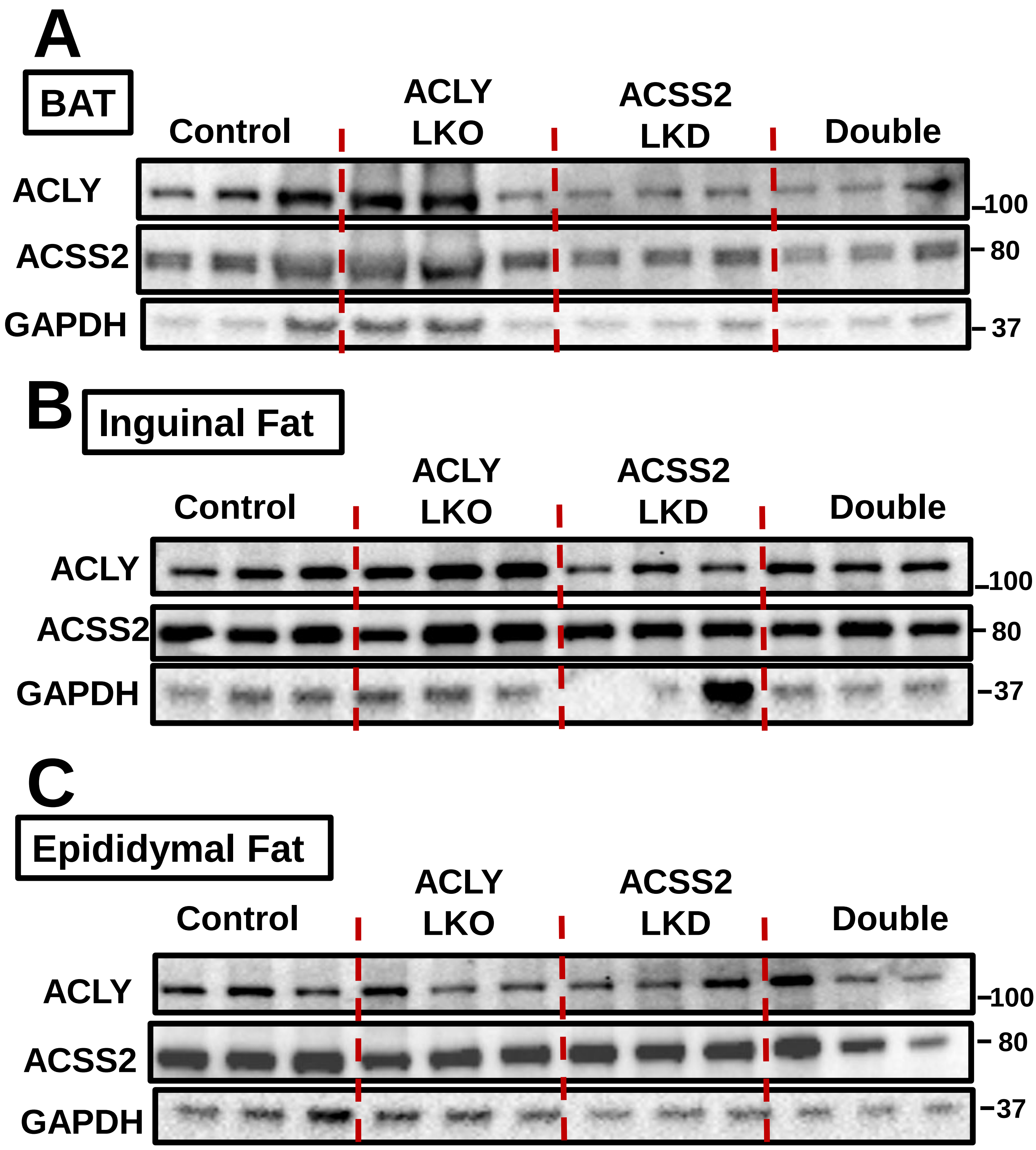

Figure S1: Confirmation of liver specific targeting of the AAV constructs: Immunoblots of ACLY and ACSS2 in **(A)** brown, **(B)** inguinal and **(C)** epididymal fat depots.

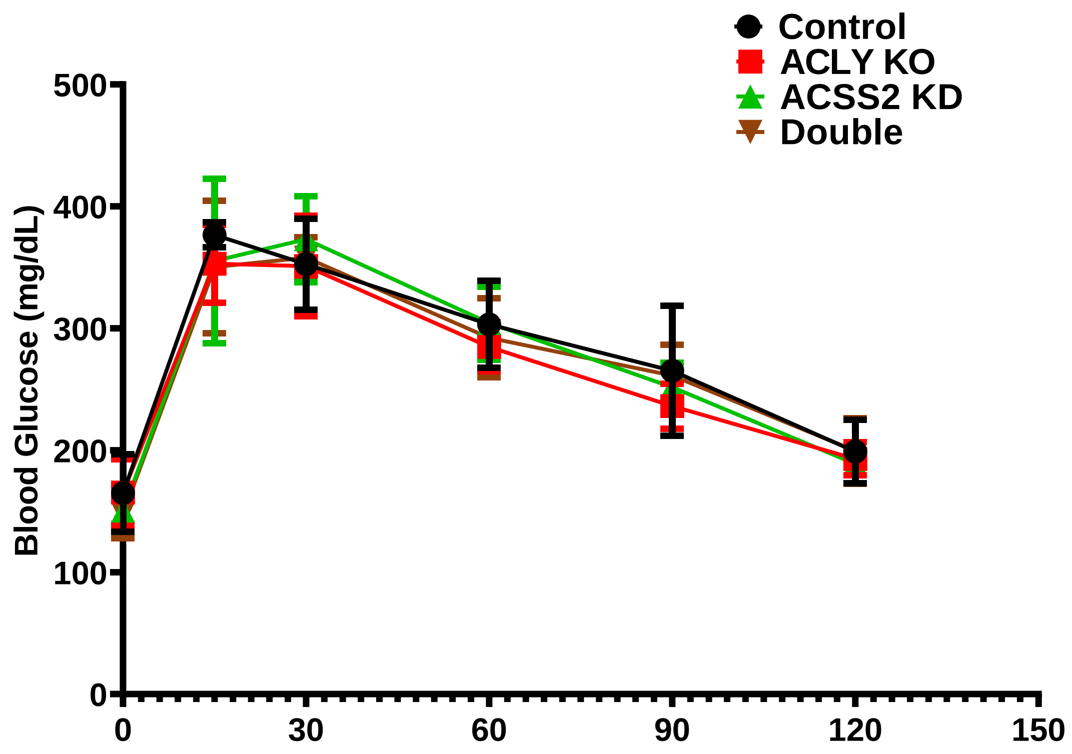

Figure S2: ACLY and/or ACSS2 depletion in HFD fed mice induced no changes in overall glucose tolerance.

A

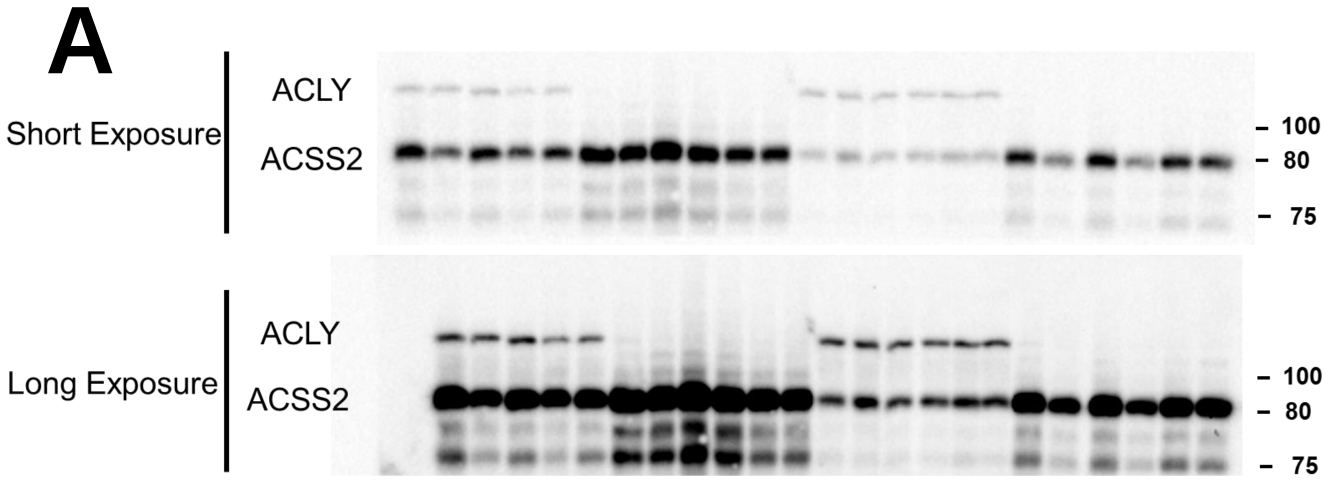

B

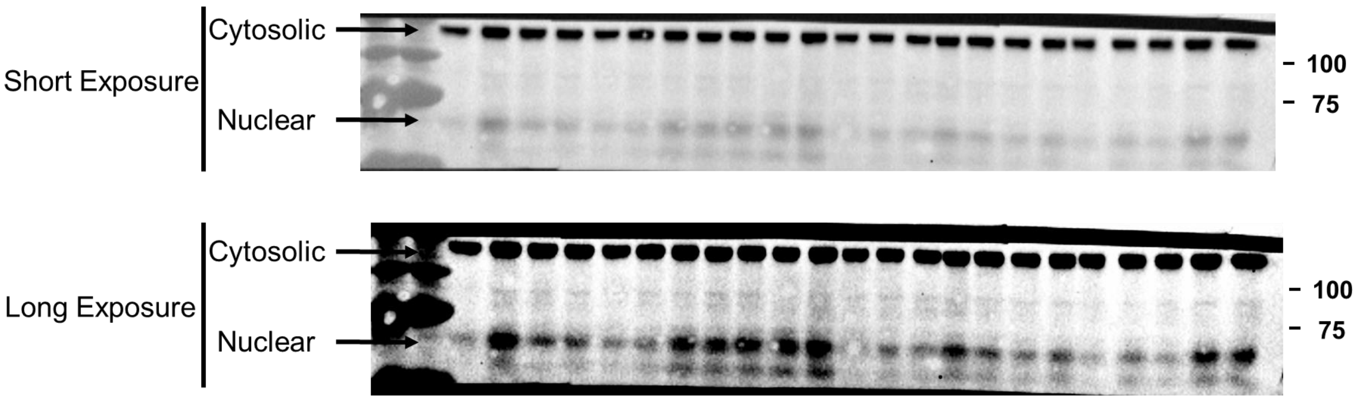

Figure S3: Raw data of (A) ACLY & ACSS2 blots in Fig. 2E and (B) SREBP1c western blots in Fig. 4B with short and long exposures

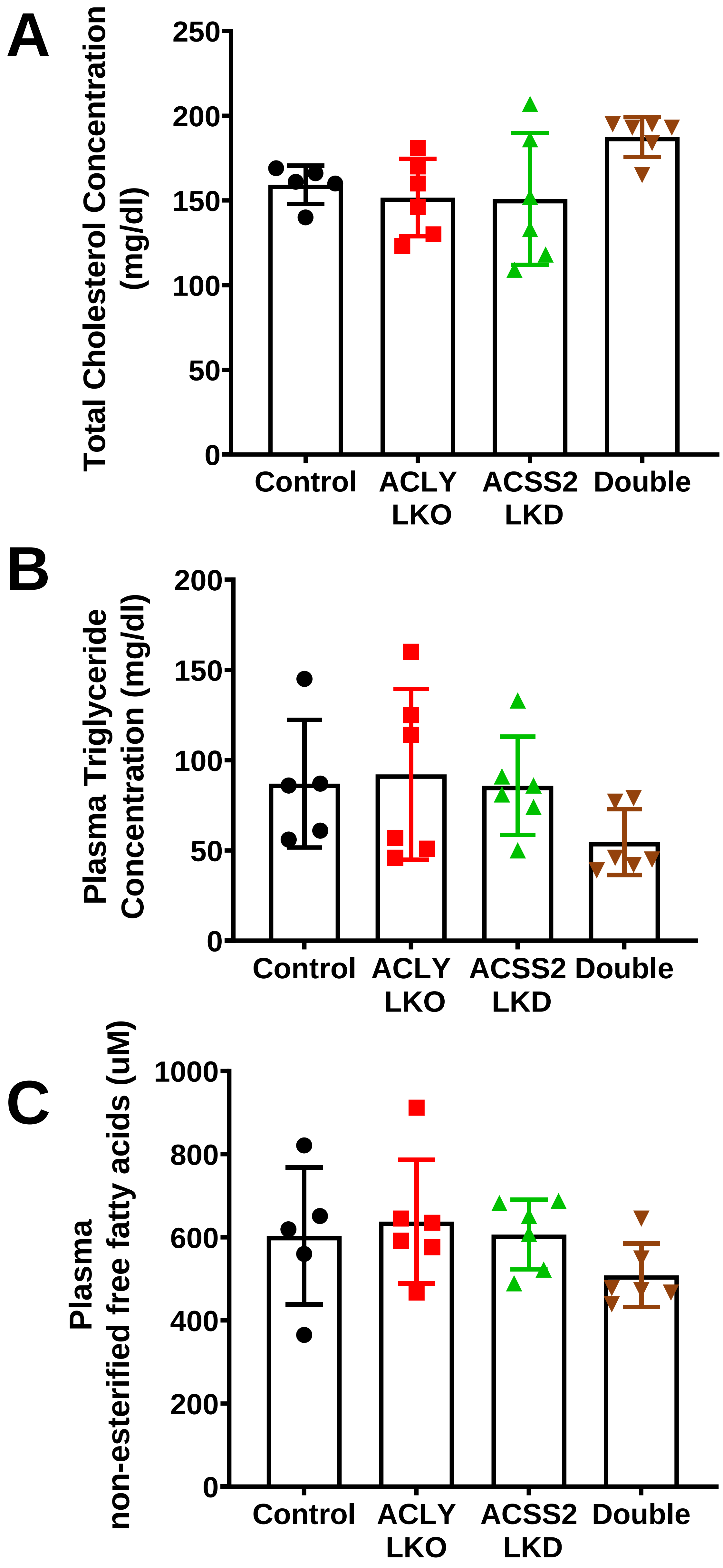

Figure S4: Liver depletion of ACLY, ACSS2 or double depletion does not change the plasma lipid profile in HFD fed mice. Plasma levels of **(A)** Total cholesterol, **(B)**TGs **(C)** Non esterified fatty acids.

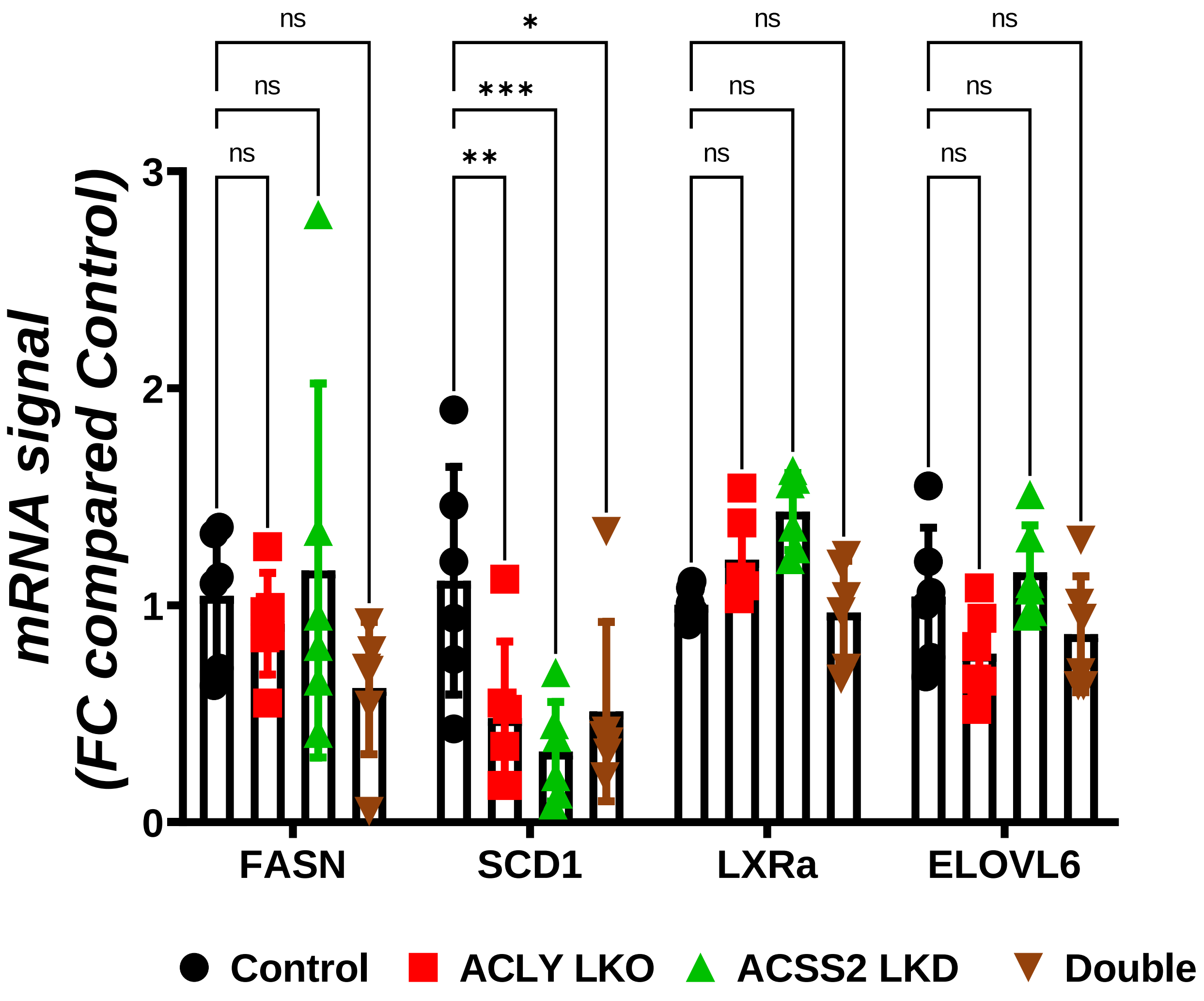

Figure S5: Liver depletion of ACLY, ACSS2 or double depletion does not induce an upregulation of DNL enzyme gene expression in CHOW fed mice.

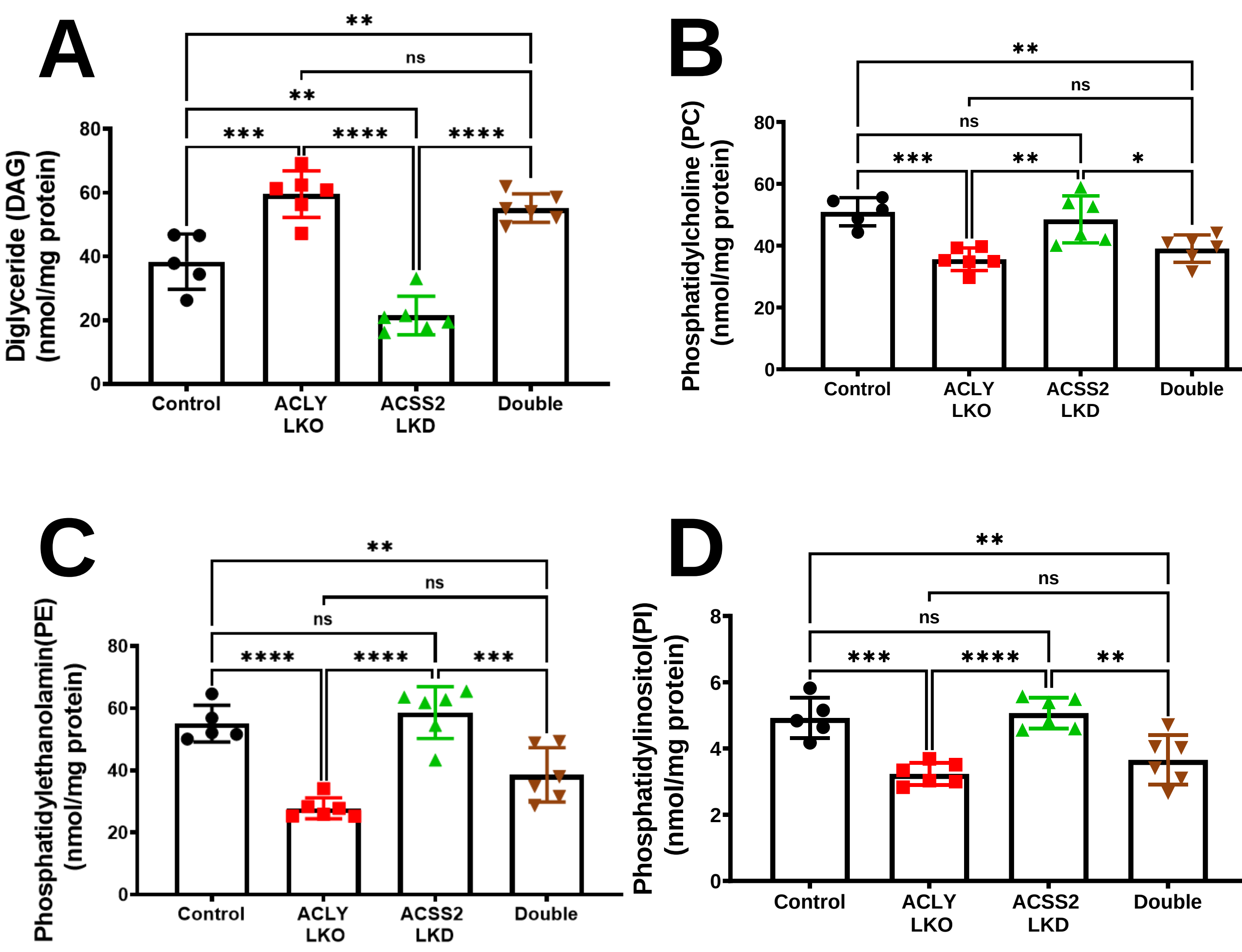

Figure S6: Lipidomics analysis demonstrates accumulation of DAGs in ACLY-depleted but not ACSS2-depleted livers, and inverse correlation with phospholipid biosynthesis. Liver **(A)** DAGs **(B)** Phosphatidylcholine **(C)** Phosphatidylethanolamine **(D)** Phosphatidylinositol levels in the livers of HFD fed groups. (ns: Not significant, *: $p<0.05$, **:$p<0.005$, ***:$p<0.0005$, ****:$p<0.00005$)

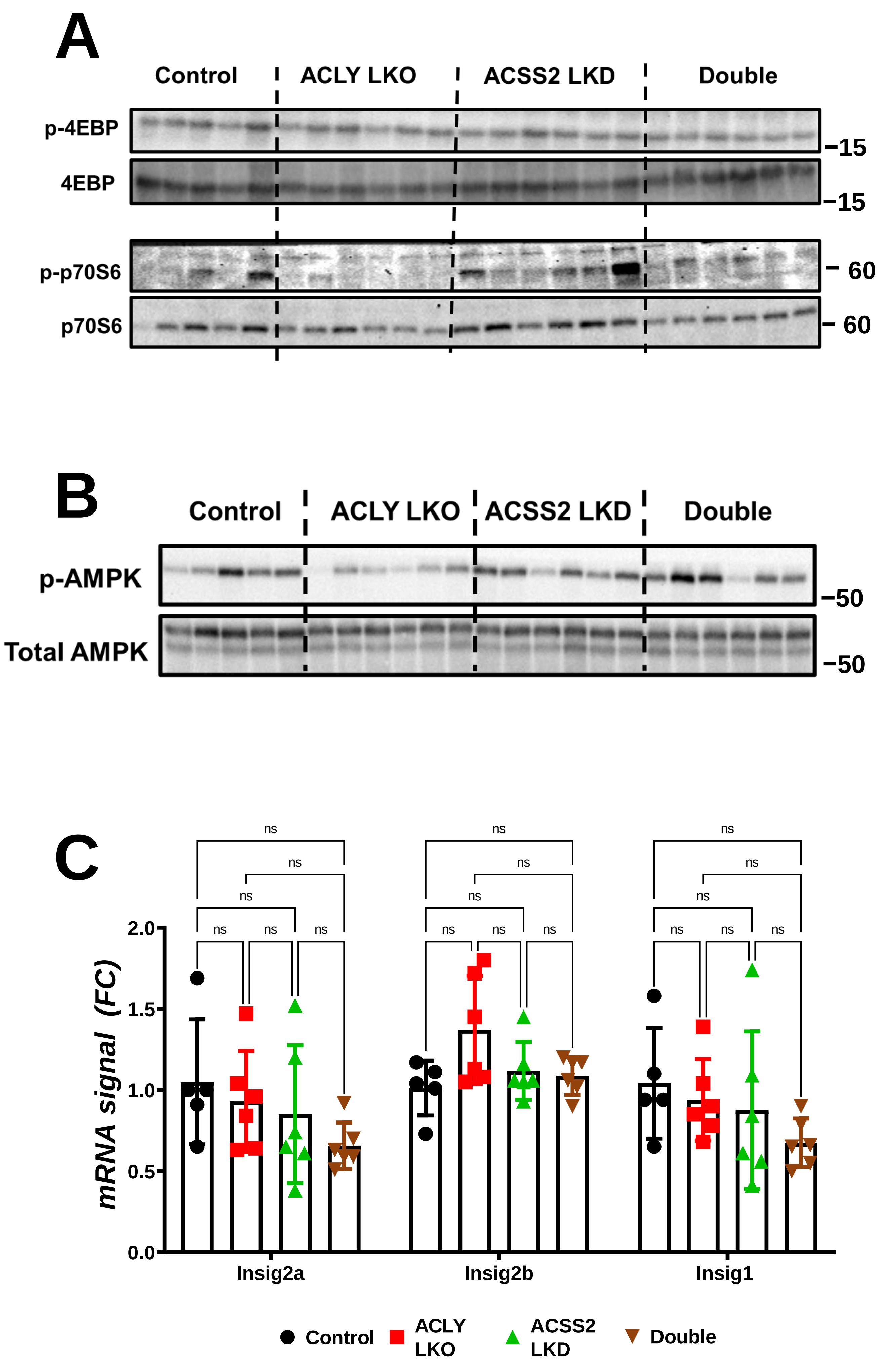

Figure S7: Increased Srebp1c processing in ACLY depleted mice in HFD is independent of (A) mTOR activation, (B) AMPK phosphorylation or (C) Insig pathways
